# The concordance of the limiting antigen and the Bio-Rad avidity assays in persons from Estonia infected mainly with HIV-1 CRF06_cpx

**DOI:** 10.1371/journal.pone.0217048

**Published:** 2019-05-24

**Authors:** Kristi Huik, Pilleriin Soodla, Merit Pauskar, S. Michele Owen, Wei Luo, Gary Murphy, Ene-Ly Jõgeda, Eveli Kallas, Heli Rajasaar, Radko Avi, Silvina Masciotra, Irja Lutsar, for the CASCADE Collaboration in the EuroCoord

**Affiliations:** 1 Department of Microbiology, Institute of Biomedicine and Translational Medicine, University of Tartu, Tartu, Estonia; 2 Clinical Retrovirology Section, HIV Dynamics and Replication Program, National Cancer Institute, National Institutes of Health, Frederick, Maryland, United States of America; 3 Laboratory Branch, Division of HIV/HIV AIDS Prevention, Centers for Disease Control and Prevention, Atlanta, Georgia, United States of America; 4 Virus Reference Department, Public Health England, London, United Kingdom; Ghent University, BELGIUM

## Abstract

**Background:**

Serological assays to determine HIV incidence have contributed to estimates of HIV incidence, monitoring of HIV spread, and evaluation of prevention strategies. Two frequently used incidence assays are the Sedia HIV-1 LAg-Avidity EIA (LAg) and the Bio-Rad avidity incidence (BRAI) assays with a mean duration of recent infection (MDRI) of 130 and 240 days for subtype B infections, respectively. Little is known about how these assays perform with recombinant HIV-1 strains. We evaluated the concordance of these assays in a population infected mainly with HIV-1 CRF06_cpx.

**Material/Methods:**

Remnant serum samples (n = 288) collected from confirmed, newly-diagnosed HIV-positive persons from Estonia in 2013 were tested. Demographic and clinical data were extracted from clinical databases. LAg was performed according to the manufacturer’s protocol and BRAI testing was done using a validated protocol. Samples with LAg-pending or BRAI-invalid results were reclassified as recent if they were from persons with viral loads <1000 copies/mL or were reclassified as long-term if presenting with AIDS.

**Results:**

In total 325 new HIV infections were diagnosed in 2013 in Estonia. Of those 276 persons were tested with both LAg and BRAI. Using assay results only, the recency rate was 44% and 70% by LAg and BRAI, respectively. The majority of samples (92%) recent by LAg were recent by BRAI. Similarly, 89% of samples long-term by BRAI were long-term by LAg. After clinical information was included in the analysis, the recency rate was 44% and 62% for LAg and BRAI, respectively. The majority of samples (86%) recent by LAg were recent by BRAI and 91% of long-term infections by BRAI were long-term by LAg.

**Conclusions:**

Comparison of LAg and BRAI results in this mostly CRF06_cpx-infected population showed good concordance for incidence classification. Our finding of a higher recency rate with BRAI in this population is likely related to the longer MDRI for this assay.

## Introduction

Estonia has the highest prevalence of HIV infections in the European Union. The HIV epidemic in Estonia likely started in 2000 among people who inject drugs (PWID) with infections mainly caused by circulating recombinant form CRF06_cpx [1, 2]. Despite a decline in the number of individuals newly diagnosed with HIV, from a total of 1,474 in 2001 to 229 in 2016, the rate of newly diagnosed infections is still high (approximately 17 per 100,000 persons in 2016) (Estonian Health Board; www.terviseamet.ee). In this situation, and outbreaks in general, the detection of recent HIV infection is important for describing the epidemic, monitoring spread, and developing and implementing important public health service interventions that prevent further HIV transmission. Thus, having a reliable incidence assay is essential for finding newly infected individuals and for assessing the efficacy of public health interventions.

Over the years, many different incidence assays have been developed. Assays are based on antibody titer [3, 4], avidity [5, 6], the proportion of HIV-1-specific immunoglobulin G (IgG) antibodies relative to total IgG [7] in human plasma and/or serum samples or a combination of these biomarkers [8, 9]. Previously, the BED-capture-enzyme immunoassay was the most commonly used assay globally [7]; however, its over-estimation of recent infections led to the development of new assays. This incidence test was called BED since it included HIV envelope antigens to subtypes B, E, and D. Now, two avidity-based assays with improved accuracy are in use–Sedia TM HIV-1 LAg Avidity EIA (LAg) [6, 10] and the Bio-Rad avidity incidence assay (BRAI), which was developed by scientists at the US Centers for Disease Control and Prevention (CDC) [11–13]. Both assays measure the binding strength (or avidity) of HIV antibodies to viral proteins, which is known to be lower in early stages of infection and increases while infection is progressing. LAg uses a multi-subtype (B, E, and D) gp41 recombinant envelope protein and BRAI uses a broader spectrum of proteins from HIV-1 Group M (subtype B) and Group O, and HIV-2 since it is a modification of an existing commercial assay for detecting HIV antibodies (Genetics Systems HIV-1/HIV-2 Plus O EIA (Bio-Rad Laboratories). In both assays, the use of a chaotropic agent is included so that less avid antibodies are dissociated and washed away, leaving only the strongly bound antibodies to be detected in the assays [6, 10–12].

Incidence assays can misclassify HIV-1-infected persons as having recent infection in individuals who have received antiretroviral therapy (ART) for long periods with viral suppression, elite controllers and persons with AIDS [13–15]. In addition, studies have shown that the performance of recency assays depends on the HIV-1 subtype, such that the mean duration of recent infection (MDRI) differs between subtypes. MDRI is a characteristic of recency assays defined as the average time spent ‘recently’ infected. For the LAg assay, the MDRI is 130 days for subtype B, 161 days for subtype A, 211 days for subtype A1, 273 days for subtype D, and one study has determined 109 days for combined samples of subtypes A and D with the normalized optical density (ODn) cut-off 1.5 [14, 16, 17]. The CDC calculated MDRI for BRAI is 240 days for subtype B using an avidity index (AI) cut-off of 30% [12, 18]. According to an evaluation by the Consortium for the Evaluation and Performance of HIV Incidence Assays, for an AI cut-off of 40%, the BRAI MDRI is the same for subtype B and C, 299 and 298 days, respectively, but longer for subtypes D and A1, 467 and 364 days, respectively [14].

Performance evaluation of incidence assays has been limited to a few non-recombinant and recombinant HIV-1 subtypes. We thus aimed to compare the performance of two frequently used HIV-1 recency assays, LAg and BRAI, in a population mostly infected with CRF06_cpx viruses. Since the LAg MDRI is shorter than that for BRAI (for subtypes for which MDRIs have been calculated), we hypothesised that if the discrepancy between the assays is solely due to differences in MDRIs then; (i) BRAI would have a higher recent infection rate than LAg; (ii) most recent infections by LAg would be recent by BRAI, and (iii) most long-term infections by BRAI would also be long-term by LAg.

## Material and methods

### Samples and demographic and clinical data

Remnant serum samples from individuals with newly-diagnosed HIV infection in Estonia between January and December 2013 were collected by the Estonian HIV Reference Laboratory. All samples were positive for HIV-1 by ELISA (HIV Ag/Ab Combo Assay; Abbott Laboratories Diagnostics Division, IL, USA or HIV Combi PT; Roche Diagnostics GmbH, Mannheim, Germany) and confirmed by Western blot (WB; NEW LAV BLOT I and NEW LAV BLOT II, Bio-Rad Laboratories Inc, Hercules, California, USA) or if WB-negative by HIV Ag and HIV Ag Confirmatory testing (both Roche Diagnostics GmbH, Mannheim, Germany) in the Estonian HIV Reference Laboratory. Serum was aliquoted and stored at -80°C until further analysis up to a maximum of two years. All study subjects were ART-naïve at the time of sampling. There were 325 newly diagnosed HIV-positive persons during the study period; 288 serum samples (88.6%) were available for LAg testing and 276 (85%) for BRAI testing. Further analyses were performed on the population that had results for both LAg and BRAI assays (N = 276). The study was approved by the Research Ethics Committee of the University of Tartu.

Demographic data (gender, age, date of diagnosis, and reason for testing) of study participants were extracted from the Estonian Health Board clinical database (Table 1). In addition, the possible date of infection, self-reported HIV transmission route, concomitant AIDS defining condition(s), HIV-1 viral load (VL), HCV serostatus, and CD4+ T cell count within six months of diagnosis were obtained from the Estonia HIV database [19]. In order to identify the last negative HIV-1 antibody test within two years prior to HIV diagnosis, the laboratory databases of four main hospitals (Tartu University Clinics, West-Tallinn Central Hospital, Narva Hospital, and Ida-Viru Central Hospital) were searched in April 2015. In addition, the date and HIV-1 VL measurements within six months of diagnosis for persons not in the Estonia-HIV database were requested from hospital laboratory databases.

**Table 1:** Characteristics of the Estonian study population tested by the incidence assays.

Variables	Study population N = 276
Male	170 (61.1%)
Median age (years)	32 (IQR: 28–41.5)
Route of transmission	
Sexual	154 (55.8%)
Parenteral	59 (21.4%)
Perinatal	1 (0.4%)
Unknown	62 (22.4%)
Median VL (log_10_ copies/ml)	4.9 (IQR: 4.4–5.6)
Median CD4+ T cells count (cells/μl)	359.5 (IQR: 205–524)
Person presenting with AIDS	13 (4.7%)
Persons with time of possible infection reported	5 (1.8%)
HCV serostatus	
Negative Positive Unknown	89 (32.3%) 79 (28.6%) 108 (39.1%)
HIV-1 subtypes	
CRF06_cpx A1 B CRF06A1 C Not determined*	172 (62.3%) 23 (8.3%) 7 (2.5%) 6 (2.2%) 1 (0.4%) 67 (24.3%)

*PCR or sequencing of the samples was unsuccessful; CRF, circulating recombinant form; CRF06A1, recombinant form between CRF06_cpx and A1; IQR, interquartile range; VL, viral load.

### HIV subtype determination

HIV-1 subtypes were determined as follows. HIV-1 pol region sequences of PR codons 24–99 and RT codons 1–235 from study subjects (GenBank accession numbers MK793369—MK793577) with subtype reference sequences from the Los Alamos HIV Sequence Database [accession numbers AF004885 (subtype A1), DQ676872 (A1), AF286238 (A2), K03455 (B), U52953 (C), K03454 (D), AF077336 (F1), AY371158 (F2), AF084936 (G), AF190127 (H), EF614151 (J), AJ249235 (K), U54771 (CRF01_AE), AY271690 (CRF02_AG), AF414006 (CRF03_AB), AB286851 (CRF06_cpx), AJ245481 (CRF06_cpx), and AF064699 (CRF06_cpx)] and recent sequences from Estonia and Russia [AY500393 (A1), AY535659 (CRF06_cpx), and DQ400856 (CRF06_cpx)] (www.hiv.lanl.gov) were used for using phylogenetic analysis (MEGA 5). The tree was rooted by the HIV-1 group O sequence (L20587). The maximum likelihood (ML) phylogenetic tree was constructed with default values (substitution model: Tamura- Nei; bootstrap replications: 500).

### Incidence assays

**Fig 1:**
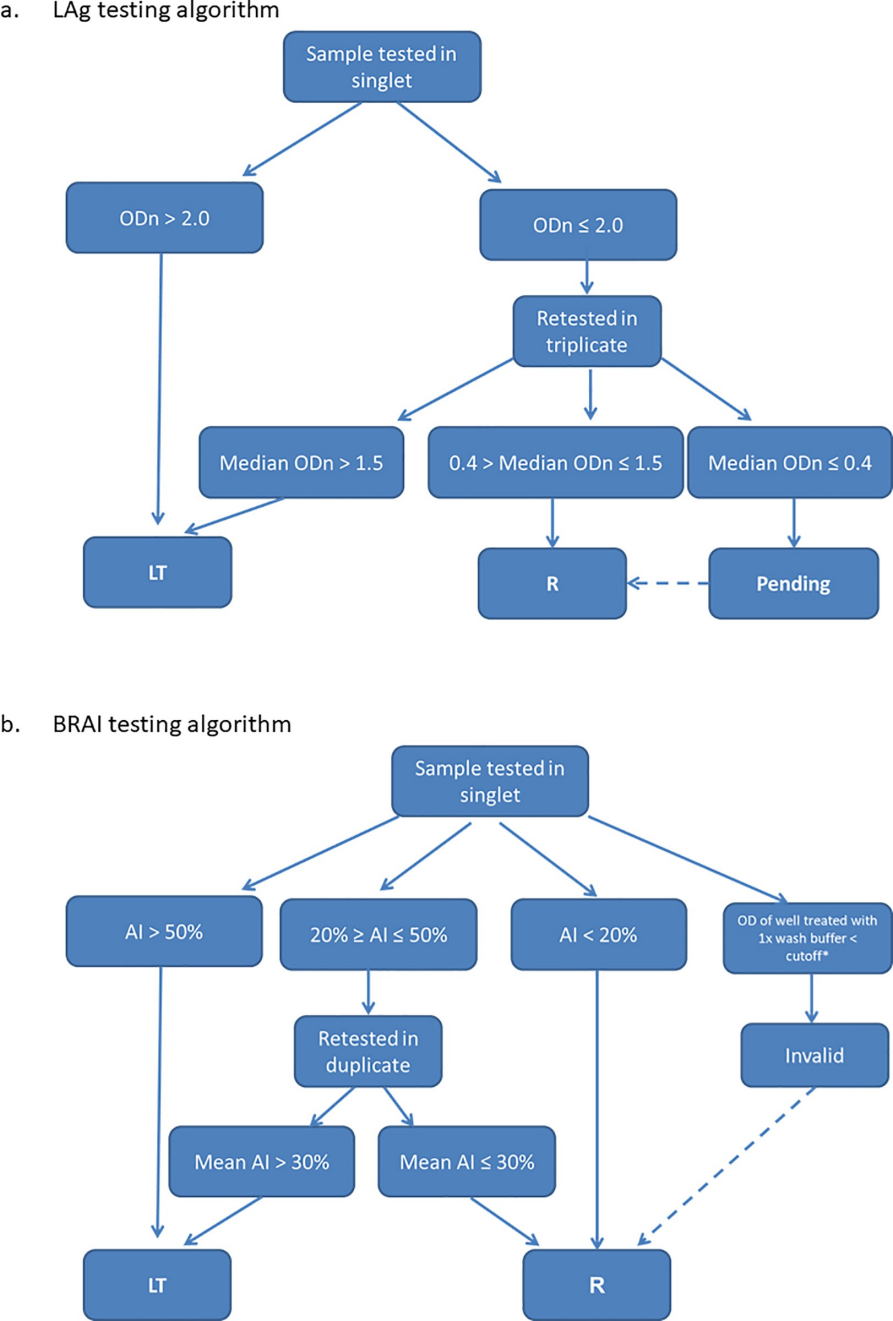
**Testing algorithms for determination of recent (R) and long-term (LT) HIV-1 infections by the (a) LAg and (b) BRAI avidity assays.** Dashed arrows in the algorithms indicate that all specimens classified as LAg-pending were reclassified as R because all samples were collected from persons confirmed to be HIV-positive and all BRAI-invalid results were reclassified as R because all samples were collected from individuals who were confirmed to have HIV infection, were treatment naïve and not classified as having AIDS. *BRAI cut-off is the mean of the optical density of the assay negative controls plus 0.250. ODn, normalized optical density.

Serum samples were first tested using the LAg assay (Sedia HIV-1 LAg Avidity EIA; Sedia Biosciences Corporation, Portland, OR, USA) according to the manufacturer’s protocol. Briefly, 0.1 M citrate buffer was used as a dissociation agent and the avidity value [normalized optical density (ODn)] was calculated by dividing the OD of sample, control, or calibrator with the median OD of the calibrator. After a first round of testing, samples with ODn value of > 2.0 were classified as long-term infection and samples with an ODn ≤ 2.0 were retested in triplicate. The retested samples with a median ODn ≤ 1.5 for the triplicate results were classified as recent infections and those > 1.5 as long-term infections (Fig 1A). Specimens with an ODn < 0.4 were considered “pending” until HIV seroreactivity was confirmed, in which case they were classified as recent if determined to HIV detectable HIV antibody.

The BRAI assay was performed according to the CDC developed protocol using the commercial Genetics Systems HIV-1/HIV-2 Plus O EIA (Bio-Rad Laboratories Inc., CA, USA) [12, 14] (Fig 1B). All samples were diluted 1:10 in a cold specimen diluent, transferred into two wells of the antigen- coated plate and incubated for one hour at 4°C. Next, either 0.1M diethylamine (DEA), a dissociation agent, or 1x wash buffer was added to separate specimen wells. After a 30 min incubation at 37° C, testing was performed as indicated in manufacture’s protocol. One each previously characterized recent and long-term serum controls were used in each plate to validate each assay run. The test plate was considered valid if (i) the recent control was below 20%, (ii) the (long-term) prevalent control was higher than 80% and (iii) both indexes were within 2 standard deviations of the mean of 20 previous runs. The optical density (OD) of the well treated with 1X wash buffer must be higher than the run cut-off (mean of OD of negative controls plus 0.250) to calculate an avidity index (AI) using the following formula: OD (DEA)/OD (wash buffer) x 100. If the OD of the well treated with 1X wash buffer was lower than the cut-off value, then the sample was considered “invalid”. Samples with an AI between 20% and 50% were retested in duplicate and the final AI is calculated as the mean of the duplicates. Samples with a final AI ≤ 30% were determined to be recent (Fig 1B). All BRAI invalid results were reclassified as recent infections if samples were collected from persons confirmed to have HIV infection, were treatment naïve (VL > 1000 copies/ml) and were not classified as having AIDS (based on the presence of opportunistic infections at the time of HIV diagnosis).

For both assays, samples with an HIV-1 VL <1000 copies/mL and those from persons presenting with AIDS were reclassified as long-term infection [13–15].

## Results

### Recency rates and assay concordance

**Fig 2:**
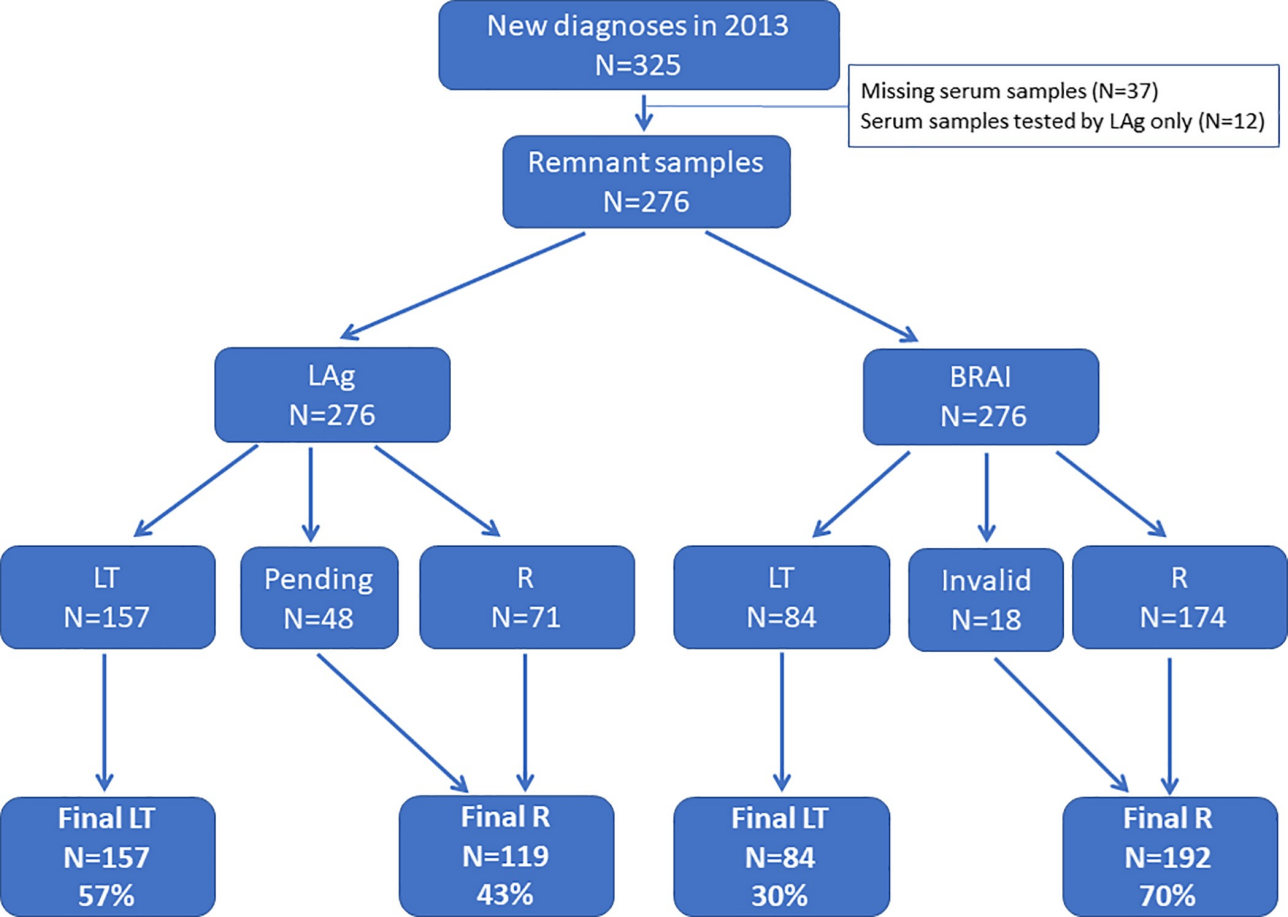
Determination of recent (R) and long-term (LT) HIV-1 infections by LAg and BRAI without inclusion of clinical data in the analysis.

Avidity assay results were first analysed without considering the clinical data in order to evaluate the direct concordance between both assays. We compared assays using 276 samples that had results of the both assays (Fig 2). Table 1 describes the demographic and clinical characteristics of the samples tested by the assays. After reclassifying specimens with LAg-pending and BRAI-invalid results with confirmed HIV infection by WB or Ag testing (see methods) as recent infections, the recency rates were 43% (119/276; 95% confidence interval [CI] 37–49%) and 70% (192/276; 95% CI 64–75%) by LAg and BRAI, respectively. The Cohen’s kappa of comparison of the assays was 0.37 (95% CI 0.28–0.47). Most LAg recent results were recent by BRAI; of 119 LAg recent, 110 were BRAI recent (92%; 95% CI 86–96%). Most samples with BRAI long-term results (84) were long-term by LAg (75) (89%; 95% CI 80–95%).

**Fig 3:**
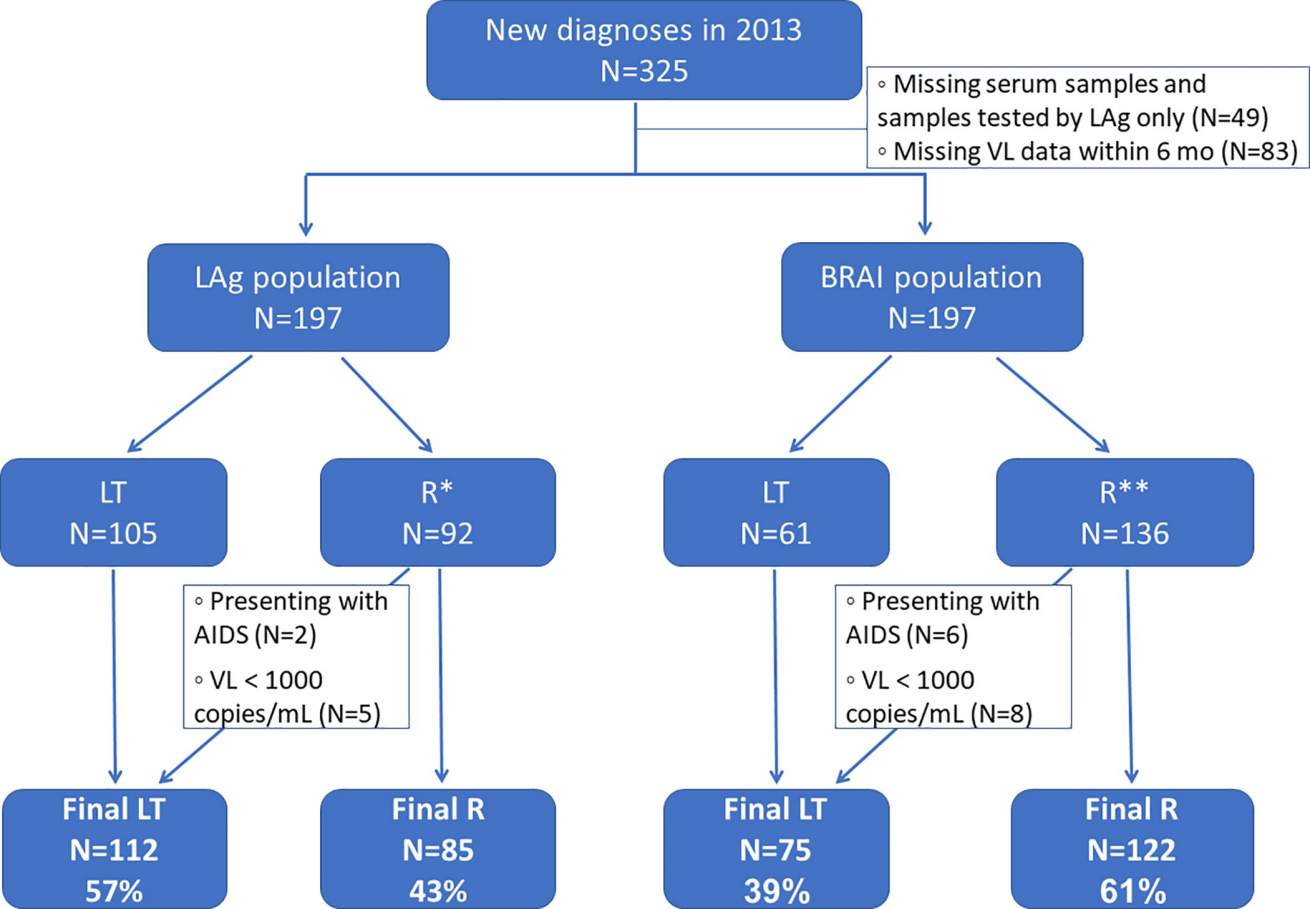
Reclassification of recent (R) and long-term (LT) infections by LAg and BRAI after including clinical data into the testing algorithm. *R by LAg includes 34 LAg-pending; **R by BRAI includes 14 BRAI-invalid samples with available viral load (VL) results performed within six months of serum collection.

Next, in order to evaluate the use of a testing algorithm for determining recency estimates, we analysed the results of samples with reported HIV-1 VL results within six months of recency testing. When taking into account HIV-1 VL and the presence of AIDS, the recency rate was similar for LAg but slightly lower for BRAI (Fig 3). There were 85 of 197 subjects (43%; 95% CI 36–50%) classified as recent infection by LAg (Fig 3). Five persons with VL <1000 copies/mL and another two presenting with AIDS were reclassified as long-term infections. For BRAI there were 122 of 197 persons (62%; 95% CI 55–69%) determined as recent infection; eight had a VL <1000 copies/mL and another six presented with AIDS and were all reclassified as long-term infections (Fig 3).

Avidity results after inclusion of clinical data were similar to those observed without inclusion of the clinical data (Figs 2 and 3). Not all LAg recent results were recent by BRAI. We found that 92% (78/85%; 95% CI 83–96%) of LAg-recent samples were recent by BRAI. Similarly, not all BRAI-long-term results were long-term by LAg; 91% (68/75; 95% CI 81–96%) of BRAI-long-term samples were long-term by LAg. An evaluation of the LAg and BRAI results among persons infected with CRF06_cpx showed that 91% (57/63; 95% CI 80–96%) of samples were recent by both assays and 83% (29/35; 95% CI 66–93%) of samples were long-term by both assays.

Next, we compared demographic and clinical characteristics between persons with recent and long-term infections in both assays separately (Table 2). We observed that persons who were LAg-recent had a higher prevalence of HCV seropositivity than those determined to be LAg- long-term. More persons with BRAI-long-term results were male compared to the BRAI-recent group. The recently infected group according to BRAI had higher CD4+ T cell counts compared to the long-term infected group (383 vs 295 cells/μL; *p* = 0.01) which was not observed for LAg. In both assays, among recently infected persons, the CRF06_cpx genotype was more prevalent than in persons with established infection. Other variables did not differ between the recent and long-term infection groups with either assay.

**Table 2:** Demographic and clinical data of persons with HIV-1 viral loads measured within 6 months after HIV diagnosis classified as long-term or recent by LAg and BRAI.

	LAg N = 197			BRAI N = 197		
	Long-term N = 112	Recent N = 85	p	Long-term N = 75	Recent N = 122	p
Male	68 (61%)	47 (56%)	0.47	**53 (70%)**	**62 (51%)**	**0.01**
Median age (years)	34 (IQR: 29–41)	31 (IQR: 26–41)	0.06	33 (IQR: 28–42)	32 (IQR: 26–41)	0.13
Route of transmission						
Sexual	68 (61%)	47 (55%)	0.31	39 (52%)	76 (62%)	0.38
Parenteral	25 (22%)	25 (30%)		21 (28%)	29 (24%)	
Unknown	19 (17%)	13 (15%)		15 (20%)	17 (14%)	
Median VL (log_10_ copies/ml)	4.9 (IQR: 4.4–5.5)	5.0 (IQR: 4.5–5.9)	0.18	5.1 (IQR: 4.3–5.6)	5.0 (IQR: 4.5–5.7)	0.42
Median CD4+ T cells (cells/μl)	334 (IQR: 189–508)	374 (IQR: 232–566)	0.15	**295** **(IQR: 148–436)**	**383** **(IQR: 238–540)**	**0.01**
R infections based on testing history* and/or possible infection date	0 (0%)	6 (7%)	NA	0 (0%)	7 (6%)	NA
HCV serostatus**						
Negative	**54 (64%)**	**29 (46%)**	**0.03**	34 (61%)	49 (54%)	0.49
Positive	**30 (36%)**	**38 (54%)**		22 (39%)	42 (46%)	
HIV-1 subtype***						
CRF06_cpx	**61 (73%)**	**63 (93%)**	**0.003**	**35 (65%)**	**89 (92%)**	**<0.001**
Other	**22 (27%)**	**5 (7%)**		**19 (35%)**	**8 (8%)**	

Significant differences are indicated in bold.

*Testing history; last negative HIV test was less than 130 days or less than 240 days prior to HIV diagnosis for LAg or BRAI, respectively.

**Persons with unknown HCV testing (n = 50) excluded

***Persons with unknown HIV subtype (n = 46) excluded; IQR, interquartile range; NA, not applicable.

### Specimens with pending and invalid results

**Fig 4:**
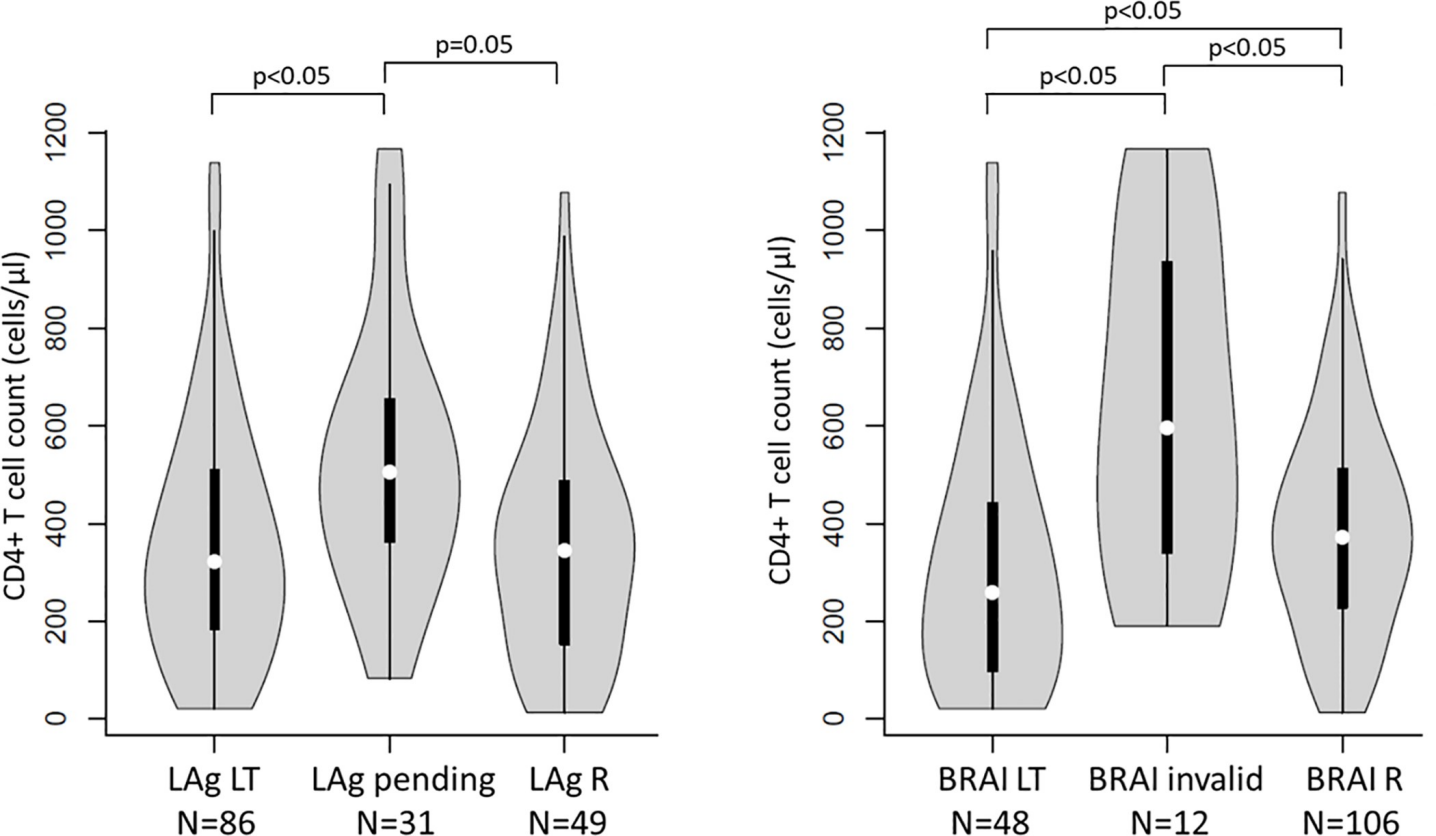
Distribution of CD4+ T cell counts between persons classified as long-term (LT), pending/invalid and recent (R) in the LAg and BRAI assays (left and right panels, respectively). The number of groups indicates the number of persons with CD4+ T cell count measurements within six months after HIV diagnosis. Only significant differences are shown.

In total, 48 samples (17%) were initially classified as pending (ODn < 0.4) by LAg and were reclassified as recent following confirmation of infection by detection of HIV antibodies or antigen. Of them, 34 (71%) had HIV-1 VL results within six months of HIV diagnosis. For BRAI, 18 samples (9%) had invalid results, including 14 (78%) with HIV-1 VL results within six months of HIV diagnosis. Of the 34 LAg-pending and 14 BRAI-invalid specimens, 31 and 12 had CD4+ T cell count measurements, respectively. Both, LAg-pending and BRAI-invalid samples had high CD4+ T cell counts with medians of 506 cells/μL (IQR 363–656 cells/μL) and 596 cells/μL (IQR 340–935 cells/μL), respectively. As shown in Fig 4, there was a difference in the median CD4+ T cell counts between the pending/invalid and long-term groups. In addition, with BRAI there was a significant difference between the invalid and recent groups. The LAg-pending group did not differ by HIV-1 VL compared to the LAg recent and LAg long-term groups (5.1 [IQR 4.6–6.4] log10 copies/ml vs 4.9 [IQR 4.2–5.7] log10 copies/ml and 5.0 [IQR 4.5–5.5] log10 copies/ml, respectively; both *p*>0.05). The BRAI-invalid group had higher HIV-1 VLs compared to the BRAI-recent and BRAI-long-term groups (6.2 [IQR 5.5–7.0] log10 copies/ml vs 4.9 [IQR 4.3–5.5] log10 copies/ml and 5.2 [IQR 4.5–5.7] log10 copies/ml, respectively; both p>0.05).

### Assay performance of samples with a reported possible infection date

Four subjects reported a possible infection date; all five persons were assumed to be infected within 111 days before HIV diagnosis (median of 74 days [IQR 69–87]) and determined to be recent infections by both assays.

Overall, previous HIV test data were available for 39/276(14%) participants; none had a prior negative test within the last 130 days, seven had negative results within 240 days and 27 (including the previous seven) had negative test results within the last two years. The distribution of these subjects according to LAg and BRAI assay final results are provided in Table 3. In total, 16/19 persons (84%) were determined as recent by both assays. Finally, we estimated the duration of infection as the midpoint between last negative and first HIV positive test and correlated these results with the avidity as measured by ODn and AI. We found no correlation between the estimated duration of infection and avidity (r = 0.31 and r = 0.30, respectively; for both p > 0.05).

**Table 3:** Distribution of subjects with a last negative HIV test prior to HIV diagnosis by LAg and BRAI testing.

	LAg analysis group^a^ Recent (n)/tested (n)	BRAI analysis group^a^ Recent (n)/tested (n)
Persons with possible date of infection	4/4 (100%)	4/4 (100%)
Last negative HIV test prior to HIV diagnosis ≤ 240 days	6/6^b^ (100%)	6/6^b^ (100%)
Last negative HIV test prior to HIV diagnosis ≤ 2 years	16/19^b^ (85%)	18/19^b^ (95%)

^a^Subjects of the results of assays after inclusion of clinical data are presented.

^b^Four persons had possible date of infection available.

## Discussion

We report the first study describing the performance evaluation of two avidity assays, LAg and BRAI, for determining incidence in a large number of patients from Estonia infected with HIV-1 recombinant form CRF06_cpx and URFs. Overall, we found high concordance between both assays for determining recency. However, not all specimens with LAg recent results were recent by BRAI and not all specimens with BRAI-long-term results were long-term by LAg, which is likely explained by the different MDRIs of each assay, with the longer MDRI for BRAI likely identifying more recent infections than the LAg assay. Using a high CD4+ T cell count as a proxy for recent infection, our results suggest that BRAI may distinguish recent and long-term infection to a greater extent than LAg in the Estonian population. In addition, our findings suggest that specimens with LAg-pending and BRAI-invalid test results with both high CD4+ T cell counts and HIV-1 VLs may truly be recent infections. These results may be specific to our study population and will require confirmation by testing of additional CRFs and non-B subtypes from Estonia and elsewhere.

Our finding that BRAI classified more specimens as recent compared to LAg supports findings from a previous report that observed longer BRAI MDRIs than those for LAg for HIV-1 subtypes A1, B, C and D [14]. Furthermore, in our analysis of MDRIs determined for subtype B for both assays, the high concordance of both recent and long-term results for both LAg and BRAI did not change when HIV-1 VL or AIDS diagnosis were taken into consideration to decrease the false recency rate (FRR) [14]. These findings likely reflect differences in MDRIs for each avidity assay that might not be the same as for subtype B. One can speculate that multiple freezing-thawing of samples might have impact on the results of the assays. However, it has been previously shown that this does not influence the performance of the assays [20]. In addition, samples tested by BRAI had only one extra freeze-thaw cycle than samples tested by LAg, but not exceeding three cycles in total. Thus, freeze-thaw cycles were unlikely to have influenced our results.

There are some possible limitations when comparing results from these two avidity assays, including their inherently different MDRIs, which have been reported to be affected by HIV-1 subtype. For example, Duong et al (2015) showed that the LAg MDRI was 122 days for subtype CRF01_AE, 152 days for subtypes C and BC and 109 days for subtypes A and D [16]. Furthermore, subtype D specimens were frequently misclassified by LAg and BRAI, likely due to weak antibody responses to HIV in subtype D infected persons over time [12, 15, 21]. Variable MDRI and FRRs among various HIV-1 subtypes (A1, B, C and D) was also reported by Kassanjee et al. (2014) where MDRIs varied from 153 to 273 days for LAg and 298 for 467 days in BRAI and the FRR ranged broadly from 0.5% to 9.1% and 2.1% to 54.5%, respectively [14]. Altogether, these reports demonstrate that avidity assay performance, characterized by MDRI and FRR, for determining incidence can be affected by antibody response to different HIV-1 subtypes and also by the composition of subtype antigens present in the assay. As our study population is predominantly infected with CRF06_cpx that do not contain subtype B-like sequences [22, 23] in the gene regions of the HIV-1 proteins utilized in the avidity assays, it is possible in our study that we may have misclassified some specimens using subtype B MDRIs. Unfortunately, MDRIs for CRF06_cpx and subtype G (CRF06_cpx consists subtype G sequence in *env* [23]) have not been estimated and would require specimen cohorts prior to ART and seroconversion panels, which are not readily available. However, our results indicate that MDRI is shorter in LAg than in BRAI.

Using high CD4+ T cell counts as a proxy for recently infected subjects, we observed higher CD4+ T cell counts in recently infected subjects compared to those with established infection by BRAI but not by LAg. Two scenarios may help explain these findings. First, the LAg assay has a shorter MDRI and may have classified many specimens as recent, which were from persons whose infections where in the phase where a transient increased drop in CD4+ T cell counts occurs prior to reaching viral set-point. During this period CD4+ T cell counts are sometimes similar between persons with recent and established infection. Alternatively, our results may suggest that the longer MDRI of BRAI may be more suitable for distinguishing recent and long-term infection in our study population. This possibility is supported by a study by Hauser et al (2014) which reported that for subtype B virus infections BRAI performed better than LAg with a true recency rate of 88% compared to 48% for LAg [12]. This assay performance difference could also be explained by the use of only gp41 envelope epitopes in LAg whereas multiple antigens are used in BRAI that can bind a broader spectrum of anti-HIV-1 antibodies and therefore might perform differently. Interestingly, the median CD4+ T cell count in persons determined to be recently infected by BRAI was relatively low (295 cells/μL) contrasting with previous studies that showed the mean CD4+ T cell count in recently infected subjects varied from 475 to 600 cells/μL [24]. Reasons for the lower CD4+ T cell counts in recently infected Estonians in our study are not well understood but are consistent with historical data in newly diagnosed patients in Estonia [25].

Only five subjects had known seroconversion dates and an additional two had negative HIV antibody tests in the previous 240 days. Samples from these persons were used as references for true recent infection and both avidity assays categorized all these subjects as recently infected. In addition, 17 of 19 infected persons had negative HIV antibody/antigen combo results during the last two years before testing positive and were also determined as recently infected by both assays. These results suggest good performance of both avidity assays in detecting recent infections. We recognize that our findings are based on a small group of patients, but recruitment of participants with known seroconversion dates is extremely difficult, especially in a population such as ours where the repeat testing rate is low (15%), likely due to a relatively high rate of HIV-1-infected PWID [25].

Surprisingly, both assays initially categorized a relatively high number of samples as pending by LAg (18%) or invalid by BRAI (9%). It is important to note that all specimens in our study were confirmed HIV-1-positive by WB or Ag testing and most of the persons also had HIV-1 RNA detected by PCR. However, specific diagnostic results were not available to determine which specimens were HIV antigen reactive only and thus acute infections. All BRAI-invalid samples were classified as recent infections, which is supported by the high HIV-1 VLs and CD4+ T cell counts of these samples. Specimens with pending/invalid avidity test results may also contain low or no antibody titers, since antibody reactivity was not detected in all of the specimens. These results may also reflect HIV-1 subtype-dependent antibody responses as suggested by Longosz et al (2014) who reported weaker avidity assay responses to HIV-1 subtype D [21]. Unfortunately, our study design did not permit any repeat specimen testing for this purpose or for comparison of avidity results between different subtypes not present in Estonia. Of note, we observed a lower rate of invalid samples in BRAI than pending samples in the LAg assay, which may be due to the broader antigen composition of BRAI, including p24 antigens which are known to be one of the first anti-HIV antibody targets following infection. In contrast, the LAg assay only contains a multi-subtype gp41 antigen. Thus, more detailed studies to further evaluate the avidity of HIV-1 antibodies to subtype CRF06_cpx infection are needed.

In summary, comparison of the LAg and BRAI avidity assays in this Estonian population infected mainly by HIV-1 CRF06_cpx viruses showed good overall concordance (>90%) in the categorization of the samples as recent or long-term infections. The different rates of recent infection are likely explained by the different assay MDRIs. Additional studies with seroconversion specimen panels from HIV-1-infected persons in Estonia are needed to further evaluate the performance of avidity assays and to determine Estonian HIV-1 subtype-specific MDRIs and FRRs.
